# Assisting document triage for human kinome curation via machine learning

**DOI:** 10.1093/database/bay091

**Published:** 2018-09-18

**Authors:** Yi-Yu Hsu, Chih-Hsuan Wei, Zhiyong Lu

**Affiliations:** National Center for Biotechnology Information, Bethesda, MD, USA

## Abstract

In the era of data explosion, the increasing frequency of published articles presents unorthodox challenges to fulfill specific curation requirements for bio-literature databases. Recognizing these demands, we designed a document triage system with automatic methods that can improve efficiency to retrieve the most relevant articles in curation workflows and reduce workloads for biocurators. Since the BioCreative VI (2017), we have implemented texting mining processing in our system in hopes of providing higher effectiveness for curating articles related to human kinase proteins. We tested several machine learning methods together with state-of-the-art concept extraction tools. For features, we extracted rich co-occurrence and linguistic information to model the curation process of human kinome articles by the neXtProt database. As shown in the official evaluation on the human kinome curation task in BioCreative VI, our system can effectively retrieve 5.2 and 6.5 kinase articles with the relevant disease (DIS) and biological process (BP) information, respectively, among the top 100 returned results. Comparing to neXtA5, our system demonstrates significant improvements in prioritizing kinome-related articles as follows: our system achieves 0.458 and 0.109 for the DIS axis whereas the neXtA5’s best-reported mean average precision (MAP) and maximum precision observed are 0.41 and 0.04. Our system also outperforms the neXtA5 in retrieving BP axis with 0.195 for MAP and the neXtA5’s reported value was 0.11. These results suggest that our system may be able to assist neXtProt biocurators in practice.

## Introduction

Document triage typically refers to the process of scanning all query-related papers and finding relevant ones for further curation. For example, in the development of biological databases such as BioGRID and UniProtKB, human curators typically first examine the results of their PubMed searches and select curatable articles based on the specific task. Given the ever-growing biomedical literature and high cost of manual curation, there is an increasing need of leveraging automatic text-mining methods to identify and prioritize the documents for manual curation. For this purpose, Critical Assessment of Information Extraction Systems in Biology (BioCreative) has recently organized several document triage challenge tasks for protein–protein interaction and Comparative Toxicogenomics Database (CTD) curation (1, 2). These efforts have resulted in several successful integration and deployment of text mining systems into production curation pipelines such as the use of PubTator and eGenPub in the UniProt protein curation (3, 4).

The ability to annotate various bioconcepts in a manually curated literature database, such as the CTD, grows to be an urgent need for retrieving entities with the highest relevance. Given the complex nature of named entities, a relevance ranking strategy for articles containing target bioconcepts to any given queries takes crucial role in building efficient search engines. Many projects in the field had adopted different tools and methods to enhance the processing of biomedical text mining. To name a few of the leading research efforts, Kim *et al*. (5) used a machine learning (ML) approach to triage CTD-relevant articles based on their prior system for the protein–protein interaction article classification task in BioCreative III. Another document triage task in BioCreative VI is the precision medicine track that aims to identify relevant PubMed citations describing mutations affecting protein–protein interactions. Fergadis *et al*. (6) proposed a bidirectional recurrent neural network, equipped with an attention mechanism and reusable sequence/document encoder architecture. The proposed system retrieves the most important elements in a sequence (6). The neXtA5 is a curation service and interface that employs different ontologies and embeds into a curation platform (7). The system assists Swiss Institute of Bioinformatics (SIB) curators to curate a given protein and axis by prioritizing relevant articles. By using various ontologies, the neXtA5 provides a better ranking of MEDLINE articles for building an advanced search engine.

**Figure 1:**
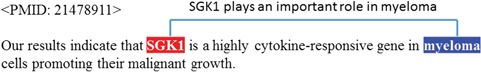
An example of positive instances in the training set. For PubMed Unique Identifier (PMID) 21478911, the extracted sentence in the abstract displays the relationship between SGK1 and myeloma as highlighted in red and blue.

Over the years, the BioCreative community has organized several tasks, aiming to bridge the gap between human curators and text-mining groups. In 2012, by examining the curation workflow of multiple databases, three important and common curation stages were identified: source collection, document triage and full curation (8). In 2017, the BioCreative VI human kinome curation track was designed specifically for providing a private data set annotated by the neXtProt team to assist both triage and annotation tasks (9). The challenge was formulated as a literature triage task that requires systems to classify and retrieve relevant articles for mentioning kinome-related biological processes (BPs) or diseases (DISs). For instance, there is a relationship between ‘Serum/glucocorticoid-regulated kinase 1 (SGK1)’ and ‘myeloma’ in Figure 1 that would be noted as <SGK1, myeloma, 21478911> relation in the task. The task organizers provided the task data based on the results of neXtProt biocurators’ routine curation processes. The human kinome curation track includes three subtasks: (i) abstract triage, (ii) full text triage and (iii) snippet selection. The task organizers (which included the neXtProt team) provided an as yet unpublished data set for the task (10).

In this work, we propose an ML approach to identify articles that describe a specific kinase and its relation to DISs or BPs in the abstract. We designed informative features to specifically capture such relationships. For instance, we compute the entity frequency and position between the kinase and related DIS/BP. Additional features are computed to further analyze the semantic relatedness of different bioconcepts. For instance, the parsing tree path can reveal a mechanism linking various bioconcepts. Finally, we designed three features to model the processes of human curation: using frequency and location features to detect the co-occurrence relation between bioconcepts and using natural language processing (NLP) features to capture the semantic information surrounding bioconcepts. Based on our evaluation, we find that our system can effectively reduce the workloads of biocurators and improve productivity.

## Methods

### Data preprocessing

BioCreative VI organizers provided two training data sets for a total of 100 proteins, including 1615 and 1844 pairs (<kinase, PMID>) and its associated axis, which can either be a BP or DIS. However, the data sets do not include which specific BP or DS associated with the annotated kinase. In this study, we combined the two sets (1615 + 1844 = 3459) and generated triples <kinase, axis, PMID>. First, we used our named entity recognizer (NER) taggers (11, 12) to recognize all kinase, DIS and BP mentions. We filtered out the articles without kinase mentions and narrowed down our results to 2775 triples. In order to evaluate our method, we kept 225 triples as a development set, leaving 2550 triples for training (Table 1). We also selected articles for 100 target proteins from 5.3 million citations, therefore creating 894 312 triples. Since there are no negative training instances provided, we generated pseudo-negatives by using the following process: first, we used a support vector machine (SVM) one-class classifier to train on the 2550 triples and tested on the 894 312 triples and then selected the lowest 2500 scores as our negative training instances (13). Note that we now have a positive set (2550 triples) and a negative set (2500 triples).

**Table 1:** Statistics of the training set. After processing, there were 3018 records in the official training set that produced 3459 triples. We further omitted articles without kinase mentions that left us 2775 triples. From this set, 2550 triples were used as training positives, and 225 triples were used as a development set

	Official	Utilized positives	
	Training set (positive instances)	Training set	Development set
# Triple	3459	2550	225
# PMID	3018	2282	221

**Figure 2:**
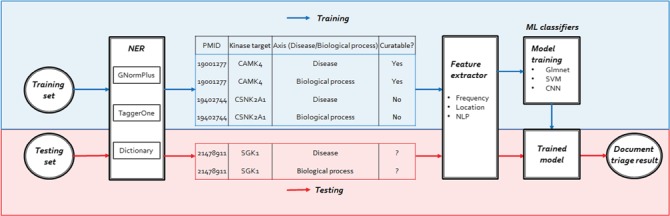
The workflow of our human kinome curation system including three steps: NER, feature extractor and training/testing.

### System architecture

Our system consists of three main components: NER, feature extractor and ML classifiers. For the NERcomponent, we annotated kinase, DIS and BP names in ML-based and dictionary approaches. After the NER component performs the document annotation, the feature extractor component would generate the following features for bioconcept entities: frequency, location and NLP features to prioritize the documents by their relevance. The ML classifiers then trained Elastic-Net Regularized Generalized Linear Models (Glmnet), SVM and Convolutional Neural Network (CNN) models to distinguish between relevant and irreverent articles. We then trained our models using different ML classifiers described in the methods section on both the 2550 positive set and 2500 negative set. After the models were built, we used the 225 triples in the development set to evaluate the ranking scores of each classifier. Figure 2 shows the overall workflow of our proposed document classification system for human kinome curation using ML.

### ML classifiers

Our submission to the abstract triage task utilizes several ML methods including Glmnet (14), SVM (15) and CNN (16). The input data for the ML-based models include only the title, abstract and bioconcept annotations of the taggers. Additionally, our methods did not distinguish between the data for DIS and BP mentions. The texts of the two types were trained together by using the same features.

### Glmnet

The features of large data sets suffer from the curse of dimensionality, and they usually generate large sparse data matrices. To reduce high-dimensional features, Glmnet is a widely used algorithm for fitting various probability distributions in statistical computing and ML. When analyzing high-dimensional data, Glmnet uses the lasso or the elastic net to interpret and fetch important features with efficient computation. Therefore, Glmnet increases in stability and makes predictions with a path of penalty parameters.

### SVM

An SVM is a robust ML algorithm for classification analysis. It has been applied to many classification problems related to supervised learning with multidimensional data. After the SVM classifier is built, the model can correctly determine the hyperplane that separates the data into different classes. We also tested one-class classification, and this model aims to find the support vectors of the one-class training set and allows for outlier/novelty detection (17). The goal is to distinguish new data as either similar or different from the normal training set. Binary classification: the original SVM is designed for determining the optimal separating hyperplane between the two groups. In practice, the SVM projects samples on a higher dimensional space to approach the optimal hyperplane with less empirical classification errors.

### CNN

A CNN is derived from deep artificial neural networks that consist of receptive fields, local connectivity and shared weights (16). The CNN has been well known for its excellent performance on image recognition. We then designed and aligned the CNN with different parameters including an input layer, convolution layer, pooling layer, fully connected layer and output layer. We carefully followed the system framework established by Kim to build the CNN model (18). In our model, each word in a sentence is represented by concatenating embeddings of its words, named entities, frequency, location, dependencies relatively to the kinase and related DIS/BP. We then applied a pre-trained model using two domain-related collections (PubMed abstracts and PMC articles) with domain-independent Wikipedia articles (19). Note that we use the following parameters for our CNN model: categorical cross-entropy; filter window sizes of 3, 4 and 5 with 300 feature maps; embedding dimensionality of 200; dropout rate of 0.5; and mini-batch size of 50.

### Features

As mentioned, we focus on three feature types concerning the processes of biocuration: using frequency and location features to detect the co-occurrence relation among bioconcepts and NLP features to capture the semantic information surrounding bioconcepts. The categorized features are shown in Table 2: (i) frequency features (features 1–2), we calculated the number of kinase and axis mentions in each abstract; (ii) location features (features 3–7), the location of kinase and axis is detected; and (iii) NLP features (features 8–11), a list of related keywords shown in Table 3. Each group includes manually generated keywords of the genetic DIS field. Furthermore, we applied tmVar (20, 21) to recognize mutation mentions in the text as an additional variation keyword group. The bag-of-words feature includes the lemma form of words around kinase, DIS and BP mentions in the abstracts. Parsing tree path features use the dependency relation of dependency grammars to record the syntactic structure of kinase, DIS and BP mentions (22). All features are transformed to document-term matrices.

**Table 2:** Statistical and linguistic features for training classifiers include the frequency of target kinase, target kinase locations and NLP information

**Feature**		**Type**
1	Number of target kinase	Numeric
2	Number of target axis	Numeric
3	Target kinase in 1st sentence	Boolean
4	Target axis in 1st sentence	Boolean
5	Target kinase in last sentence	Boolean
6	Target axis in last sentence	Boolean
7	The same sentence	Boolean
8	Kinase keywords	String
9	Bag of words	String
10	Parsing tree path	String
11	Parsing tree path w/o ancestors	String

**Table 3:** The keyword groups include related terms that are often employed in the articles to describe kinases, DIS and BP, such as verb, patient, genetic and scale

Group	Keywords
Verb	involve, enhance, inhibit, regulate, increase, associate, phosphorylate
Patient	patient, men, women
Genetic	detectable, survival, genetic, tumorigenesis, overexpression, mutation, translate, transcript, change, lymphangiogenic, neurotrophic
Scale	mg, kg
Period	day(s), during
Examine	examine, experiment, screen, role, risk, significant
Variation	recognized by tmVar

## Result

Before submitting official runs, we used the following evaluation metrics to assess each of our classifiers: mean average precision (MAP) is the mean of the precision scores for various queries, and it evaluates the retrieval results that represent the average of the precisions (denoted as AP) for the set of queries. Using the MAP calculations, an article was ranked as curatable if it associated with kinome-related information. In an information retrieval system, precision (P) is the fraction of retrieved documents that are relevant to the query:

(1) \begin{equation*} \mathrm{P}=\frac{\left|\left\{ relevant\ documents\right\}\cap \left\{ retrieved\ documents\right\}\right|}{\left|\left\{ retrived\ documents\right\}\right|}\end{equation*}

When we consider only the top *k* documents returned, the precision value can be evaluated at fixed levels of retrieved results, known as P@K. AP is the average of precision values after relevant documents are retrieved. A precision score of zero would be designated to those where relevant documents are not retrieved. AP can be defined as the following equation:

(2) \begin{equation*} \mathrm{AP}=\frac{\sum_{i=1}^kP@i\times rel(i)}{\left|\left\{ relevant\ documents\right\}\right|} \end{equation*}

where $i$ is the rank, $rel\left(\right)$ is an indicator function on the relevance of a given rank, and $P@i$ is the precision at a top $i$ documents. Note that the value of $rel(i)$ equals to 1 when the item at rank $i$ is a relevant document; otherwise, the value would be 0. We first calculated the sum of AP for individual query $q$. MAP score is the mean of APs on the set of queries $Q$. MAP is defined as follows:

(3) \begin{equation*} \mathrm{MAP}=\frac{\sum_{q=1}^Q{AP}_q}{\mid \!Q\!\mid } \end{equation*}

We also defined an estimated score ($Escore;\mathrm{see}\ \mathrm{below}$) for measuring the ranking result of a triple (*t*) including a kinase, an axis and a PMID. We trained the model with the training set (positives: 2550 triples and negatives: 2500 triples), and then we added the development set (225 triples) to all the triples derived from the 100 target proteins. Note that ${\gamma}_t$ is the rank of a triple after we combined the triples of the training and development set ($\mid\!\!{D}_t\!\!\mid$). We then summarized the score $\frac{\gamma_t}{\mid{D}_t\mid }$ of all 225 triples where $\mid\!\!\!{t}_D\!\!\!\!\mid$ represents the total number of triples in the development set. For example, if we assume there are 10 PMIDs mentioned for a target kinase and the rank of one specific PMID is the top one among all 10 PMIDs, then $\frac{\gamma_t}{\mid{D}_t\mid }$ is 0.1. Therefore, the lower ${Escore}_t$ represents a better performance.

(4) \begin{equation*} {Escore}_t=\sum_{\mid{t}_D\mid}\frac{\gamma_t}{\mid {D}_t \mid } \end{equation*}

For official results, the organizers used the following evaluation metrics. P10 is the precision at rank 10: it is calculated using the number of documents that are relevant among the top 10 documents returned by a system. If a system returns 10 documents and only 5 documents are relevant, the P10 is 0.5. Similarly, P30 and P100 are the precision at rank 30 and 100, respectively. R30 is the recall at rank 30: it is calculated using the number of relevant documents retrieved in the top 30 documents returned by a system. For instance, let us say for each query, there are only 20 relevant documents. If a system returns five of these relevant documents in its top 30 results, then the R30 score is 0.25. Similarly, R100 is the recall at rank 100. P at R0 is the maximum precision observed at all ranks. That is, the recall with the highest precision is R0. R-Prec is the precision observed at rank r, where r is the number of relevant documents in the collection. If a given query contains 20 relevant documents, R-Prec is the precision at rank 20.

As shown in Table 4, we trained different models with features described in Table 2. For the Glmnet classifier, both BP and DIS triples are included in the training set. An SVM (binary) is the model in which we applied both positives and negatives as a binary classifier, while an SVM (one class) uses only the positives to train a one-class classifier. For the CNN classifiers, we constructed multiple hidden layers between the input and output layers and modeled complex nonlinear relationships. The evaluations of different methods on training sets (including DIS and BP sets) are reported in Table 5. Overall, the performance of the Glmnet classifiers is superior compared to the other two classifiers. After reviewing and optimizing the parameters in the training set, we then used the following methods and features in Table 4 as our 10 submitted runs.

**Table 4:** Combinations of different methods and features used in the training phase to obtain models for specific classifiers

Method	Features	Positives of training set	Classifier
1	9	2550	Glmnet
2	1–9	2550	Glmnet
3	1–10	2550	Glmnet
4	1–9, 11	2550	Glmnet
5	1–10	2550	SVM (binary)
6	1–9, 11	2550	SVM (binary)
7	1–9, 11	2550	SVM (one class)
8	1–9, 11	2550	CNN

**Table 5:** Evaluation of training set. We adopted the aforementioned development set to assess the outcomes after applying the training set to eight separate models

Method	$ Escore $	MAP
1	58.09	0.0401
2	49.84	0.0535
3	46.46	0.0593
4	49.85	0.0598
5	52.88	0.0460
6	57.32	0.0470
7	82.20	0.0227
8	83.68	0.0204

Tables 6 and 7 demonstrate the official results of our submitted runs (provided by the task organizers). Note that KinDER is the other participating team (23) and neXtA5 (7) is the benchmark system. In addition to the eight models in Table 5, we built two new models using the entire 2775 training pairs with Glmnet (#9) and CNN (#10). Both tables show that Glmnet classifiers have higher performance in MAP than that of SVM and CNN classifiers, which is consistent with our observation in the training and development phases. When considering our own $Escore$ metrics, Glmnet classifiers are also consistent with the best performance compared to all classifiers.

**Table 6:** Official results of kinases/DIS for the 10 submitted runs. Comparing with Kinder and neXtA5, method 3 (Glmnet) outperformed in the categories of MAP and P at R0

**Method**	**MAP**	**R-Prec**	**P at R0**	**P10**	**P30**	**P100**	**R30**	**R100**
3 (Glmnet)	0.109	0.147	0.458	0.152	0.098	0.052	0.222	0.327
9 (Glmnet)	0.109	0.145	0.453	0.148	0.097	0.052	0.223	0.327
4 (Glmnet)	0.108	0.142	0.455	0.151	0.098	0.052	0.225	0.326
5 (SVM_Binary_)	0.088	0.125	0.351	0.119	0.081	0.044	0.203	0.304
6 (SVM_Binary_)	0.088	0.125	0.351	0.117	0.081	0.044	0.201	0.304
1 (Glmnet)	0.081	0.098	0.370	0.103	0.075	0.042	0.184	0.286
7 (SVM_One class_)	0.079	0.099	0.338	0.103	0.075	0.042	0.182	0.288
2 (Glmnet)	0.073	0.084	0.338	0.094	0.064	0.038	0.166	0.269
8 (CNN)	0.062	0.079	0.224	0.075	0.054	0.036	0.150	0.265
10 (CNN)	0.060	0.079	0.227	0.065	0.057	0.034	0.154	0.259
KinDER	0.098	0.134	0.370	0.138	0.087	0.047	0.196	0.304
neXtA5	0.040	–	0.410	–	–	–	–	–

**Table 7:** Official results of kinases/BP for the 10 submitted runs. Method 3 (Glmnet) performed better than neXtA5 in MAP, whereas KinDER performed slightly better than Method 3 in the categories of MAP and P at R0

**Method**	**MAP**	**R-Prec**	**P at R0**	**P10**	**P30**	**P100**	**R30**	**R100**
3 (Glmnet)	0.195	0.182	0.450	0.176	0.121	0.065	0.399	0.563
9 (Glmnet)	0.192	0.184	0.430	0.171	0.122	0.064	0.397	0.563
4 (Glmnet)	0.191	0.178	0.437	0.171	0.122	0.064	0.396	0.561
5 (SVM_Binary_)	0.172	0.168	0.379	0.143	0.107	0.057	0.361	0.526
6 (SVM_Binary_)	0.170	0.169	0.378	0.140	0.107	0.057	0.361	0.524
1 (Glmnet)	0.159	0.150	0.379	0.138	0.105	0.057	0.362	0.535
2 (Glmnet)	0.155	0.141	0.373	0.137	0.104	0.056	0.346	0.529
8 (CNN)	0.127	0.109	0.251	0.086	0.074	0.045	0.292	0.468
7 (SVM_One class_)	0.119	0.109	0.242	0.101	0.077	0.046	0.285	0.457
10 (CNN)	0.109	0.078	0.219	0.075	0.064	0.044	0.266	0.455
KinDER	0.201	0.210	0.466	0.184	0.111	0.056	0.360	0.492
neXtA5	0.110	–	0.450	–	–	–	–	–

## Discussion and conclusion

We applied various statistical and linguistic features to prioritize the abstracts with relationships between target kinase and DIS/BP mentions. However, this task is, by nature, very challenging. First, there are no gold-standard ‘non-curatable’ or negative documents provided in the training set. Without such negative data, most classification methods have difficulty identifying truly curatable articles. Second, the relationships between DIS/BP mentions and the target kinases are not clearly curated in the training set. For example, one abstract may have multiple DIS/BP mentions. Therefore, it is difficult to find the correct triples for feature extraction in our method. Third, the low-recognition rate of GNormPlus missed ∼20–40% of the kinases (60% of the missed kinases are not in the abstracts). Finally, according to the official results, our best performance reached a P100 = 0.052, R100 = 0.327 in kinases/DIS and P100 = 0.065, R100 = 0.563 in kinases/BPs that compares favorably to the other participating team. This also means that for each target kinase, the average numbers of curatable articles are 15.90 and 11.55, respectively, that are extremely low concerning the millions of articles in PubMed. Thus, we suspect that human curators may only include articles with strong evidence within the experimental results section. Therefore, those negative articles discarded by human curators cannot be filtered by our methods as they currently use only information from titles and abstracts. For these reasons, this task is much more difficult than many other traditional document classification tasks.

In summary, we used several ML methods with frequency, location and NLP features for the neXtProt triage task that aims to specifically retrieve PubMed articles with biomedical relations among kinases, DIS and BPs. The average number of curatable articles in the testing set is low (15.90 of target kinases/DISs articles and 11.55 of target kinases/BPs articles). Thus, the biocurators using our system can retrieve 32.7% (5.2 articles) and 56.3% (6.5 articles) of curatable articles among all PubMed articles after reviewing only 100 articles returned by our system. Therefore, we believe our system can effectively accelerate the manual curation efforts. In future work, we plan to examine the triage task based on full texts as well as investigate a robust ML approach that is capable of using curatable labeled data only.
